# Combined Mastectomy and Laparoscopic Hysterectomy with Salpingo-Oophorectomy in Transgender Men: a Cohort Study

**DOI:** 10.1007/s43032-021-00724-x

**Published:** 2021-10-05

**Authors:** Daniela Gold, Marie-Christine Bertholin y Galvez, Christian Laback, Riccarda Hartleb, Gordana Tomasch, Stephanie Schöpfer, Vesna Bjelic-Radisic, Rüdiger Hochstätter, Gunda Pristauz-Telsnigg, Rene Laky, Philipp Reif, Arnim Bader, Karl Tamussino

**Affiliations:** 1 Department of Gynecology and Obstetrics, Medical University of Graz, Auenbruggerplatz 14, 8036 Graz, Austria; 2 Division of Plastic, Aesthetic and Reconstructive Surgery, Medical University of Graz, Graz, Austria

**Keywords:** Gender affirming surgery, Gender dysphoria, Transgender, Complications

## Abstract

There are few data on complications with gender affirming surgery. The aim of this study was to assess peri- and postoperative complications of laparoscopic hysterectomy and mastectomy performed in a single sitting in transgender men. Assessment of intra- and postoperative complications in a series of 65 transgender men (mean age 27, range 18–47) undergoing concomitant mastectomy and laparoscopic hysterectomy with salpingo-oophorectomy. Mean operating time was 292 ± 47 min. Thirty-four (52%) patients experienced complications: 28 (41%) DINDO grade I, 0 DINDO grade 2, 6 (11%) DINDO grade III. The six grade 3 complications consisted of 5 hematomas requiring evacuation after mastectomy and 2 vaginal tears requiring transvaginal repair. Three patients were readmitted within 30 days, all for postoperative bleeding/hematoma. In transgender men, performing laparoscopic hysterectomy and mastectomy at a single sitting has a modest rate of perioperative complications, and may improve resource utilization.

**Supplementary Information:**

The online version contains supplementary material available at 10.1007/s43032-021-00724-x.

## Introduction

The incidence of gender dysphoria is approximately 0.6% and many of these persons will seek medical care to align their gender identity with their physical characteristics [1]. Treatment for transgender men includes hormone therapy with testosterone and gender affirming surgery (GAS) [1]. Transgender men can undergo mastectomy, hysterectomy, salpingo-oophorectomy, colpectomy, and phalloplasty [2]. Few studies have addressed postoperative outcomes after mastectomy or hysterectomy in transgender men [3–8]. Complication rates seem to be low for both procedures; however, only few studies have reported data on transgender men undergoing laparoscopic hysterectomy and concomitant mastectomy [9–11].

The present study analyzed intra, peri-, and postoperative adverse events in transgender men undergoing laparoscopic hysterectomy and mastectomy in a single setting. We hypothesized that combined GAS has a modest rate of adverse events.

## Methods

This was a retrospective cohort study. Consecutive patients from 2007 onwards to 2020 undergoing mastectomy and hysterectomy in a single sitting were included in the analysis. Inclusion criteria were female to male- gender dysphoria confirmed by a psychological or psychiatric assessment and age between 18 and 50 years; exclusion criteria were not applicable.

Demographic variables such as age, BMI, parity, smoking, and comorbidities were extracted from hospitals records. Operative reports and medical records were reviewed to assess intraoperative complications and any adverse events including bleeding, injury to organs, wound infection, deep vein thrombosis, pulmonary embolisms, and 30-day readmissions. Complications were graded according to the DINDO classification [12].

Mastectomies were performed in a subcutaneous fashion by a plastic surgeon or a gynecologist with a single incision in the lower half of the mammary gland. A free nipple graft was done if necessary, based on breast size or ptosis of the breast. All patients received a compression bandage of the chest for 6 weeks.

Laparoscopic hysterectomy with bilateral salpingo-oophorectomy was performed using 4 trocars and bipolar vessel sealing. Our technique entails opening the retroperitoneum to identify the ureter. The vagina was closed with delayed absorbable sutures. Postoperatively, heavy physical activity was not recommended; otherwise, no restrictions were imposed.

Two and 6 weeks postoperatively patients were asked to return for a check-up visit.

All patients received thromboembolic prophylaxis with low-molecular-weight heparin perioperatively.

Institutional ethics committee approval was obtained (EK-number: 30–087 ex 17/18).

Statistical analyses were done with Excel. Only demographic variables were calculated using mean and standard deviation or frequency and percent.

## Results

Between 2007 and 2019, 65 transgender men underwent mastectomy and laparoscopic hysterectomy with bilateral salpingo-oophorectomy as a combined procedure at our institution. Demographic characteristics of the patients are shown in Table 1. Thirty-four patients (52%) underwent simple mastectomy, and 31 (48%) had mastectomy with a free nipple transplant. Mean total operating time was 292 ± 47 min (range 175–389).

**Table 1:** Demographic characteristics of 65 transgender men undergoing hysterectomy and mastectomy

	M (SD) or n (%)
Age (y)	27 (7)
BMI	23.8 (6.2) (range 18–38)
Diabetes mellitus	1 (1.5%)
Smoking	59 (91%)
Psychological disorder	14 (22%)
Highest education (*n* = 34)	**n**
No finished school	2 (6%)
Compulsory schooling	9 (26%)
Vocational school	15 (44%)
High school	2 (6%)
University/college	5 (15%)
Relationship status (*n* = 25)	**n**
Single	18 (72%)
In a relationship	7 (28%)

*y* years, *m* median, *SD* standard deviation, *n* frequency

Thirty-four patients had 35 complications (28 DINDO 1, 7 DINDO III) (Table 2). The 7 DINDO III complications were 5 post-mastectomy hematomas requiring surgical evacuation and 2 vaginal tears requiring transvaginal suturing. No patients required blood transfusion, and there were no recorded cases of thromboembolic events or compartment syndrome.

**Table 2:** Peri- and postoperative complications in 65 transgender men undergoing mastectomy and hysterectomy

	n	DINDO class
Mastectomy-related complications		
Seroma	8 (12%)	I
Nipple areola complex necrosis	0	
Wound infection	3 (5%)	I
Haematoma	11 (17%)	I
Haematoma requiring reoperation	5 (8%)	IIIb
Hysterectomy-related complications		
Urinary tract infection	4 (6%)	I
Vaginal tear requiring reoperation	3 (5%)	IIIb
Postoperative voiding dysfunction	1 (1%)	I
Vaginal cuff bleeding	1 (1%)	I
Deep venous thrombosis /PAE	0	
Transfusion rate	0	
Neurologic deficits	0	
Compartment syndrome	0	

*n* frequency, *class* classification, *PAE* pulmonary artery embolization

One patient had transient voiding dysfunction with increased post void residual (up to 150 ml) which resumed within 3 months of operation without any further intervention.

The 30-day readmission rate was 5% (*n* = 3); one patient had bleeding from a vaginal tear needing surgical revision 3 weeks postoperatively, one patient had oozing from the vaginal cuff with no further treatment, and one patient had bleeding from the breast 5 days postoperatively needing surgical evacuation.

The mean follow-up period was 3 ± 1.9 years (min 4 months, max 12 years). Three patients had liposuction after mastectomy; 44 had scarring in the breast with 24 (55%) undergoing scar revision, respectively. One patient underwent scar revision surgery twice.

## Discussion

Our study indicates low rates of complications in transgender men undergoing mastectomy and hysterectomy in a single sitting. The most common reason for reintervention were hematomas or bleeding.

Four studies have addressed outcomes with combined surgery and all have indicated that combining laparoscopic hysterectomy and mastectomy in a single sitting does not appear to increase the rate of complications [9–11, 13]. Cizek et al. reported no breast-related complications and two major complications related to hysterectomy in a series of 25 patients [9]. One patient had intraabdominal bleeding after vaginal hysterectomy of a fibroid uterus requiring uterine artery embolization, and the other had a partial vaginal cuff dehiscence. Vaginal cuff dehiscence has been reported to vary between 0.12 and 0.7% in ciswomen depending on type of hysterectomy and occurs mainly after postoperative sexual intercourse [14, 15]. In our study, we did not have this issue, probably due to the low number of patients having vaginal intercourse [2].

The transgender team from the Netherlands also reported few major complications in a series of 32 patients with combined surgery; there was one conversion from laparoscopy to Pfannenstiehl due to a dermoid cyst, and one patient with both breast and vaginal hematoma needing punctation [10].

Elfering et al. [11] reported the largest series to date with 212 patients. This group reported postoperative hematoma of the chest in 16% of patients which is similar to our results. Reoperation for hematoma was done in 7.5% in patients with mastectomy only while this was the case in 16% in the combined group without an obvious reason for the higher reoperation rate in the combined procedure [11]. In our cohort, however, surgical evacuation for hematoma was needed in only 8%.

Similarly, Mayrhofer et al. [13] found breast hematoma to be the most common cause of postoperative complications in 16.7% of patients in the second largest series published.

The mastectomy technique for transgender men differs from mastectomy in ciswomen and is known to cause more hematomas than conventional mastectomy because it aims to eliminate the female aspect of the breast by removing both glandular tissue and excessive skin and many require a free nipple graft [3].

Even though most study patients smoked, few complications occurred, especially in regard to free nipple grafts. It seems that the free nipple graft is often useful for mastectomy in transgender men. The low complication rate in our cohort is in line with a study by Knox et al., who also saw few complications with this surgical technique in contrast to the concentric circular technique [16].

Secondary corrections of the breast for scarring problems have been reported to range from 9 to 46%. On average, a third of patients undergoes revision which is similar in our cohort [3]. These are cosmetic procedures, not reinterventions for complications.

Laparoscopy appears to be the approach of choice for hysterectomy in transgender men. It is minimally invasive and affords access to the adnexa in patients who are often nulliparous. Our study and others suggest that laparoscopic hysterectomy in transgender men is not associated with more complications than in ciswomen [6]. The reoperations were due to vaginal tears incurred during extraction of the uterus through the vagina. Nulliparity and testosterone-induced atrophy probably predispose to vaginal tears in this setting. There were no ureteral injuries in our series.

A strength of our study is that it is one of the few reports on postoperative outcome after GAS both for mastectomy and hysterectomy in a single sitting and the second largest so far. Also, in the Austrian healthcare system billing, issues between surgical disciplines are not an issue. Limitations are the lack of subjective outcome data, the moderate cohort size, and the lack of a control group.

The low incidence of complications can be explained by patients characteristics; they are young with a low rate of comorbidities. Furthermore, laparoscopic hysterectomy is a standard gynecological procedure.

## Conclusions

Our results suggest that combined laparoscopic hysterectomy and mastectomy is a good option for transgender men requiring GAS, despite operating times averaging almost 5 h. Performing mastectomy and hysterectomy with salpingo-oophorectomy in a single sitting has modest peri- or postoperative complications and may improve resource utilization.

## Supplementary Information

Below is the link to the electronic supplementary material.

Supplementary file1 (DOCX 13 KB)

## Data Availability

The data are not publicly available.

## Code Availability

Not applicable.

We wish to disclose that many of the patients in the present series went on to have colpectomy and urethral lengthening. This data are published in the International Journal of Transgenderism (Voiding dysfunction after colpectomy and urethral lengthening in transgender men).
