# Supplementary material for: Combined Mastectomy and Laparoscopic Hysterectomy with Salpingo-Oophorectomy in Transgender Men: a Cohort Study
Source: Reprod Sci. 2021 Oct 5;28(12):3515–8. doi: 10.1007/s43032-021-00724-x (PMC8580897; doi:10.1007/s43032-021-00724-x)
Supplement: Supplementary file 1 — Supplementary file1 (DOCX 13 KB) [file 43032_2021_724_MOESM1_ESM.docx]

| **Author** | **Breast hematoma requiring surgical evacuation** | **Vaginal hematoma / tear requiring surgical evacuation** | **Hysterectomy related complications** | **Vaginal cuff dehiszence** |
| --- | --- | --- | --- | --- |
| Gold et al. | 5/65 (8%) | 4/65 (6%) | Postoperative voiding dysfunction 1/65 (1%) | 0 |
| Cizek et al. | 0 | 0 | Uterine artery embolization 1/25 (4%) | 1/25 (4%) |
| Ott et al. | 1/32 (3%) | 1/32 (3%) | Conversion from laparoscopy to Pfannenstiehl 1/32 (3%) | 0 |
| Elfering et al. | 23/212 (10.8%) | 2 (0.8%) | Reoperation for abscess 1 (0.4%) | 1 (0.4%) |
| Mayrhofer et al. | 5 (4.6%) | 3 (1.8%) | Conversion to laparotomy 2 (1.9%) | 0 |
